# Heat Shock Gene Inactivation and Protein Aggregation with Links to Chronic Diseases

**DOI:** 10.3390/diseases6020039

**Published:** 2018-05-18

**Authors:** Ian James Martins

**Affiliations:** 1 Centre of Excellence in Alzheimer’s Disease Research and Care, Sarich Neuroscience Research Institute, Edith Cowan University, Verdun Street, Nedlands 6009, Australia; i.martins@ecu.edu.au; Tel.: +618-6304-2574; 2 School of Psychiatry and Clinical Neurosciences, The University of Western Australia, Nedlands 6009, Australia; 3 McCusker Alzheimer’s Research Foundation, Hollywood Medical Centre, 85 Monash Avenue, Suite 22, Nedlands 6009, Australia

**Keywords:** immunogenic proteins, heat shock proteins, amyloid beta oligomers, temperature regulation, autoimmune disease, Sirtuin 1, heat shock factor 1, mitophagy, NAFLD, neurodegenerative diseases

## Abstract

The heat shock response involved in protein misfolding is linked to the formation of toxic immunogenic proteins with heat shock proteins (HSP) as regulators of amyloid beta aggregation. The defective amyloid beta trafficking between different intracellular compartments is now relevant to HSPs and autoimmunity. Overnutrition, temperature dysregulation, and stress repress the heat shock gene Sirtuin 1 with the induction of HSP regulated amyloid beta aggregation involved in the autoimmune response. Defective circadian rhythm alterations are connected to inactivation of the peripheral sink amyloid beta clearance pathway and related to insulin resistance, protein aggregation, and autoimmune disease in non-alcoholic fatty liver disease (NAFLD) and various neurodegenerative diseases such as Alzheimer’s disease. Nutritional therapy is critical to prevent immunosenescence, and plasma Sirtuin 1 levels should be determined to reverse, stabilize, and prevent protein aggregation with relevance to mitochondrial apoptosis and programmed cell death in chronic diseases.

## Commentary

Major interests in the heat shock response with relevance to disturbed protein homeostasis of heat shock proteins (HSP) are relevant to various chronic diseases [1]. Transcription factors such as the mammalian family of heat shock factors (HSFs) are proteins that under gene regulation are involved with protein misfolding [2,3,4] and the formation of toxic immunogenic proteins such as HSP that regulate the amyloid beta oligomer formation [5,6] associated with various human diseases [7,8]. The identification of the heat shock gene Sirtuin 1 (Sirt 1) a NAD^+^ dependent class III histone deacetylase is involved with the prevention of insulin resistance and with the deacetylation of HSF 1 is identified to protect neuron cells from proteotoxicity and cell death in chronic and neurodegenerative diseases [9,10]. Sirt 1 under temperature regulation has become important as a transcriptional regulator of HSF 1 [10] to protect neurons from protein-damaging stress associated with misfolded proteins such as HSP 70–induced amyloid beta peptide oligomer formation and with HSP 70–regulated insulin receptor complexes.

**Figure 1:**
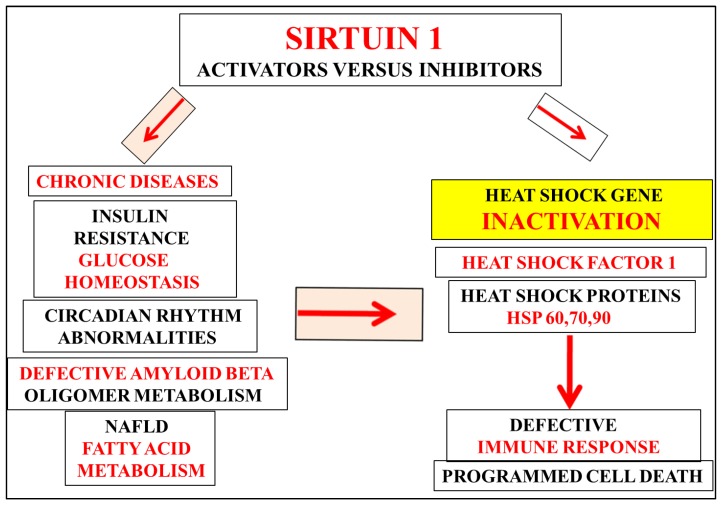
Unhealthy diets are involved with defective Sirt 1 function that determine proteotoxicity and cell death in chronic and neurodegenerative diseases. Temperature dysregulation will inactivate the heat shock gene Sirt 1 and the therapeutic role of Sirt 1 activators that determine circadian rhythm disorders, insulin resistance, non-alcoholic fatty liver disease (NAFLD). Heat shock gene inactivation and the defective immune response is connected to the immunogenic heat shock proteins (HSP) and amyloid beta oligomers with relevance to programmed cell death.

Overnutrition, temperature dysregulation, and stress [11,12,13] are associated with Sirt 1 dysregulation and connected with induction of pro-inflammatory cytokines and autoimmune disease [14]. Sirt 1 and its relevance to autoimmune disease [15] may involve the dysregulation of immunogenic HSPs and amyloid beta oligomers associated with cell apoptosis (Figure 1). Under stress conditions, Sirt 1 dysregulation [12] is important to immunotherapeutics, and its repression may be the primary defect in HSPs and amyloid beta misfolding with defective protein trafficking between different intracellular compartments now relevant to these immunogenic proteins in various diseases such as obesity, diabetes, non-alcoholic fatty liver disease (NAFLD), neurodegenerative diseases, and Alzheimer’s disease [12].

**Figure 2:**
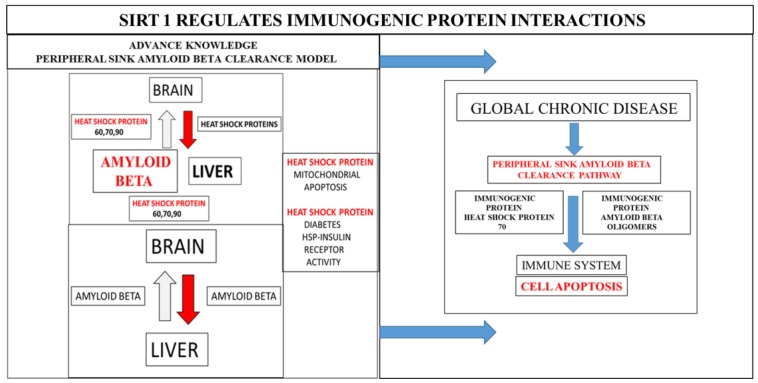
In the peripheral sink amyloid beta clearance pathway experiments from cell culture and in vivo studies indicate from many laboratories that monomeric amyloid beta is cleared from the brain to the liver. Temperature dysregulation and unhealthy diets will corrupt the peripheral amyloid beta clearance pathway linked to immunogenic HSP mediated amyloid beta aggregation relevant to mitophagy, cell apoptosis and autoimmune disease in global chronic disease.

Sirt 1 is a calorie sensitive gene [11,16,17] and its repression by overnutrition and bacterial lipopolysaccharides (LPS) interfere with the peripheral sink amyloid beta clearance pathway [11,17,18,19]. Inappropriate nutrient intake (excess glucose and fatty acids) involves repression of Sirt 1 that inactivates the immune system [15] with defective immunogenic proteins (Figure 2) related to circadian rhythm disorders, immunosenescence, and programmed cell death [15,20,21,22,23]. Temperature dysregulation and stress inactivate the heat shock gene Sirt 1 involved with suprachiasmatic nucleus (SCN) function and linked to whole body glucose and fatty acid homeostasis connected to the immune response (Figure 1 and Figure 2) in various species and man [12,13,15,20,21,22,23]. In various diseases such as diabetes, NAFLD, and various chronic diseases, the SCN defect [10,12] is the primary defect with Sirt 1 defective circadian regulation of cellular HSPs now relevant to insulin resistance and autoimmune disease and global chronic diseases [15,20,21,22,23] (Figure 1).

Nutritional therapy that include Sirt 1 activators determine neuron proliferation [24], with Sirt 1 post-transcriptional dysregulation connected to circadian rhythm disturbances, insulin resistance, and NAFLD [11,12,16]. Sirt 1 activators include leucine, pyruvic acid, α-lipoic acid, resveratrol, magnesium, zinc, pyrroloquinoline quinone, and rutin [25], and Sirt 1 inhibitors include excess palmitic acid, excess butyric acid, alcohol, suramin, sirtinol, arginine, LPS, patulin, and various xenobiotics. The effects of LPS with relevance to Sirt 1 repression supersedes Sirt 1 activators with relevance to its role in membrane transformation and as a competitive inhibitor of Sirt 1 in the induction of circadian rhythm abnormalities, insulin resistance and NAFLD [11,12,18,19]. In the developing world elevated LPS are associated with Sirt 1 dysregulation [19] with autoimmune disease associated with mitochondrial apoptosis, insulin resistance linked to various chronic diseases [15]. In the global NAFLD epidemic, the critical interest in immunotherapy [15,20] that involves Sirt 1 as the primary treatment may be relevant to stabilization of NAFLD associated chronic diseases.

Ineffective immunobiotherapy with heat shock gene Sirt 1 inactivation [15] may interfere with immunisation and vaccine-preventable diseases essential for maintenance of various chronic diseases. Nutritional therapy and immunotherapeutics involve the measurement of plasma Sirt 1 levels [26,27,28] that determine immunogenic proteins levels and mitochondrial function in various chronic diseases. Diets that activate the heat shock gene Sirt 1 are required to increase Sirt 1 levels in the plasma and brain [28] to prevent programmed cell death. Sirt 1 activators versus Sirt 1 inhibitors [25] may determine protein misfolding with the formation of toxic immunogenic proteins that induce acute cell death associated with various human diseases (Figure 1 and Figure 2). Plasma Sirt 1 levels and HSP [8,9,10,15,17] early in life may be critical to the reversal, stabilization, and prevention of various chronic diseases, with measurement of Sirt 1 [26,27,28] essential to accompany various diagnostic tests for immunological and chronic diseases [15].

## References

[1] Kakkar V., Meister-Broekema M., Minoia M., Carra S., Kampinga H.H. (2014). Barcoding heat shock proteins to human diseases: Looking beyond the heat shock response. Dis. Models Mech..

[2] Vabulas R.M., Raychaudhuri S., Hayer-Hartl M., Ulrich F. (2010). Protein Folding in the Cytoplasm and the Heat Shock Response. Cold Spring Harb. Perspect. Biol..

[3] Cantó C. (2017). The heat shock factor HSF1 juggles protein quality control and metabolic regulation. J. Cell Biol..

[4] Gomez-Pastor R., Burchfiel E.T., Thiele D.J. (2018). Regulation of heat shock transcription factors and their roles in physiology and disease. Nat. Rev. Mol. Cell Biol..

[5] Dalgediene I., Lasickiene R., Budvytyte R., Valincius G., Morkuniene R., Borutaite V., Zvirbliene A. (2013). Immunogenic properties of amyloid beta oligomers. J. Biomed. Sci..

[6] Maier M., Seabrook T.J., Lazo N.D., Jiang L., Das P., Janus C., Lemere C.A. (2006). Short amyloid-beta (Abeta) immunogens reduce cerebral Abeta load and learning deficits in an Alzheimer’s disease mouse model in the absence of an Abeta-specific cellular immune response. J. Neurosci..

[7] Kaul G., Thippeswamy H. (2011). Role of Heat Shock Proteins in Diseases and Their Therapeutic Potential. Indian J. Microbiol..

[8] Jindal S. (1996). Heat shock proteins: Applications in health and disease. Trends Biotechnol..

[9] Martins I.J. (2016). Heat shock gene Sirtuin 1 regulates post-prandial lipid metabolism with relevance to nutrition and appetite regulation in diabetes. Int J. Diabetes Clin. Diagn..

[10] Martins I.J. (2016). Type 3 diabetes with links to NAFLD and Other Chronic Diseases in the Western World. Int. J. Diabetes.

[11] Martins I.J. (2015). Overnutrition Determines LPS Regulation of Mycotoxin Induced Neurotoxicity in Neurodegenerative Diseases. Int. J. Mol. Sci..

[12] Martins I.J. Nutritional Diets Accelerate Amyloid beta Metabolism and Prevent the Induction of Chronic Diseases and Alzheimer’s Disease. https://www.researchgate.net/publication/279533471_Nutritional_diets_accelerate_amyloid_beta_metabolism_and_prevent_the_induction_of_chronic_diseases_and_Alzheimer’s_disease.

[13] Regulation of Body Temperature and NAFLD in Global Population. Feb 2018. Asian Hospital and Healthcare Management e-Newsletter. https://www.asianhhm.com/.../regulation-of-body-temperature-and-nafld-in-global-po.

[14] Martins I.J. (2017). Defective Interplay between Adipose Tissue and Immune System Induces Non Alcoholic Fatty Liver Disease. Updates Nutr. Disorders Ther..

[15] Martins I.J. (2018). Appetite Control and Biotherapy in the Management of Autoimmune Induced Global Chronic Diseases. J. Clin. Immunol. Res..

[16] Martins I.J. (2016). Diet and Nutrition reverse Type 3 Diabetes and Accelerated Aging linked to Global chronic diseases. J. Diabetes Res. Ther..

[17] Martins I.J. (2017). Calorie Sensitive Anti-Aging Gene Regulates Hepatic Amyloid Beta Clearance in Diabetes and Neurodegenerative Diseases. EC Nutr..

[18] Martins I.J. (2015). Unhealthy Diets Determine Benign or Toxic Amyloid Beta States and Promote Brain Amyloid Beta Aggregation. Austin J. Clin. Neurol..

[19] Martins I.J. (2017). The Future of Genomic Medicine Involves the Maintenance of Sirtuin 1 in Global Populations. Int. J. Mol. Biol..

[20] Martins I.J. (2018). Biotherapy and the Immune System in Ageing Science. Acta Sci. Nutr. Health.

[21] Labrecque N., Cermakian N. (2015). Circadian Clocks in the Immune System. J. Biol. Rhythms..

[22] Habbal O.A., Al-Jabri A.A. (2009). Circadian rhythm and the immune response: A review. Int. Rev. Immunol..

[23] Scheiermann C., Kunisaki Y., Frenette P.S. (2013). Circadian control of the immune system. Nat. Rev. Immunol..

[24] Martins I.J. (2016). Early diagnosis of neuron mitochondrial dysfunction may reverse global metabolic and neurodegenerative disease. GJMR.

[25] Martins I.J. (2017). Nutrition Therapy Regulates Caffeine Metabolism with Relevance to NAFLD and Induction of Type 3 Diabetes. J. Diabetes Metab. Disorders.

[26] Martins I.J. (2018). Sirtuin 1, a Diagnostic Protein Marker and its Relevance to Chronic Disease and Therapeutic Drug Interventions. EC Pharmacol. Toxicol..

[27] Martins I.J. (2018). Evaluation of diagnostic tests in human health and disease. J. Clin. Pathol. Lab. Med..

[28] Martins I.J. (2017). The Limitations of Food Intake and Biomarkers in the Prevention of Chronic Diseases. Nov. Technol. Nutr. Food Sci..

